# Effectiveness of active video games for promoting physical activity: an umbrella review

**DOI:** 10.3389/fspor.2025.1706145

**Published:** 2025-11-17

**Authors:** Víctor Juan Vera-Ponce, Jhosmer Ballena-Caicedo, Fiorella E. Zuzunaga-Montoya, Carmen Inés Gutierrez De Carrillo

**Affiliations:** 1 Facultad de Medicina (FAMED), Universidad Nacional Toribio Rodríguez de Mendoza de Amazonas (UNTRM), Amazonas, Perú; 2 Universidad Continental, Lima, Perú

**Keywords:** video games, exercise, motor activity, physical fitness, systematic review

## Abstract

**Introduction:**

Active video games (AVG) have emerged as a potential strategy to combat global physical inactivity, transforming sedentary screen time into physical activity. However, the evidence on their effectiveness remains fragmented and heterogeneous.

**Objective:**

To synthesize the available evidence from systematic reviews on the effectiveness of active video games for promoting physical activity in different populations.

**Methods:**

An Umbrella Review (UR) was developed following PRIOR guidelines. Six databases were searched until April 2025. Systematic reviews that evaluated AVG and physical activity were included. Methodological quality was assessed with AMSTAR 2 and certainty of evidence with GRADE. Two independent reviewers performed selection and data extraction.

**Results:**

Twenty systematic reviews were included encompassing 418 unique primary studies with >180,000 participants. The most studied platforms were Nintendo Wii (16 reviews), Xbox Kinect (11 reviews), Dance Dance Revolution (8 reviews) and Pokémon Go (3 reviews). AVG consistently achieved light-moderate intensity (3–6 metabolic equivalents or METs) during gameplay. The overall effect was moderate (Hedges g = 0.525, 95%CI: 0.322–0.728) but with high heterogeneity (*I*^2^ > 75%). Older adults showed the most consistent benefits [effect size (ES) = 0.64–0.68 muscle strength; ES = 0.79 cardiorespiratory fitness] with high certainty. Only 23% of interventions showed sustained post-intervention effects. Methodological heterogeneity was extreme: different metrics such as METs, moderate-vigorous physical activity (MVPA), steps/day; instruments (accelerometers, calorimetry, questionnaires) and protocols (single sessions to 48 weeks). Only 30% of reviews systematically reported adverse events.

**Conclusions:**

AVG are effective for promoting physical activity during their use, especially in older adults and overweight individuals. However, the lack of methodological standardization makes it impossible to establish specific recommendations. Consensus on measures and protocols is urgently required to realize the potential of AVG as a public health tool.

## Introduction

Active video games (AVG), also known as exergames, represent an interactive technology category that combines digital entertainment with physical activity, requiring body movements to control gameplay (1). These platforms—including Nintendo Wii, Microsoft Kinect, PlayStation Move, and augmented reality applications like Pokémon GO—have evolved substantially since their commercial introduction in the mid-2000s, incorporating motion sensors and body-tracking technology that demand active participation (2). AVGs can be classified into commercial off-the-shelf (COTS) exergames, mass-market entertainment products adapted for physical activity (e.g., Wii Sports, Just Dance), and serious games, specifically designed with health promotion objectives (3). COTS exergames offer accessibility and intrinsic motivation through engaging gameplay, while serious games can incorporate evidence-based behavioral change techniques, though often facing engagement challenges.

In a global context where insufficient physical activity constitutes a leading mortality risk factor—responsible for approximately 5 million annual deaths according to recent WHO estimates (4)—AVGs have been proposed as an innovative intervention strategy. It is critical to distinguish between sedentary behavior (low energy expenditure while sitting/reclining) and insufficient moderate-to-vigorous physical activity (MVPA), defined as failing to meet WHO recommendations of 150 min weekly for adults or 60 min daily for children (4, 5). Recent surveillance data indicate that children in high-income countries may spend 8-10 h daily in front of screens, while over 80% of adolescents globally do not meet MVPA guidelines (6, 7). AVGs uniquely occupy an intermediate space by transforming screen-based activities into forms achieving light-to-moderate intensity physical activity, typically ranging from 3 to 6 metabolic equivalents (METs) during gameplay, comparable to brisk walking (8, 9). However, intensity varies considerably by game genre: dance and boxing games generate higher energy expenditure than balance-focused or upper-limb games, and these MET values reflect session intensity rather than changes in habitual physical activity patterns.

The unique value of AVGs lies in overcoming traditional physical activity barriers through: immediate feedback and gamification enhancing intrinsic motivation, reduced perceived exertion potentially improving adherence, accessibility for individuals with mobility limitations or environmental barriers, and scalability through home-based implementation (2, 10). Applications have been investigated across diverse settings: schools as complements to physical education, homes as alternatives to sedentary entertainment, clinical contexts for chronic condition management, and community spaces through location-based augmented reality (8). Each setting presents distinct advantages—schools offer population access but face time constraints; home programs allow flexibility but suffer higher attrition; clinical settings provide monitoring but raise sustainability questions beyond supervised contexts. However, this versatility has resulted in considerable methodological heterogeneity in outcome measures and intervention protocols, creating a fragmented evidence base. Additionally, sparse systematic reporting of adverse events—including cybersickness in virtual reality modalities, fall risk in older populations, and musculoskeletal injuries—limits comprehensive risk-benefit assessments.

Despite growing numbers of systematic reviews, notable inconsistencies persist regarding AVG effectiveness. Evidence ranges from reviews reporting moderate-to-large positive effects to others finding minimal or transitory effects not persisting beyond intervention periods. Given rapid technological evolution—characterized by platform obsolescence, emerging modalities (virtual reality, mixed reality), and shifting market dynamics—periodic high-level syntheses are essential to map the evidence landscape. Furthermore, existing reviews exhibit significant methodological variability and inconsistent quality assessments, making it difficult for stakeholders to draw definitive conclusions.

Therefore, the present umbrella review aims to synthesize and critically evaluate available evidence from systematic reviews on AVG effectiveness for promoting physical activity. Importantly, this review focuses specifically on direct physical activity measures (METs, MVPA, daily steps) rather than functional rehabilitation outcomes (balance, gait, coordination), which represent distinct therapeutic targets with different evaluation frameworks. Through rigorous analysis, this study seeks to quantify aggregate AVG effects, identify factors contributing to result variability, examine critical gaps including safety reporting, and provide evidence-based recommendations for clinical practice, public health policy, and future research directions.

## Methodology

### Study design

An umbrella review (UR) was conducted following the methodological guidelines of the Joanna Briggs Institute (JBI) for reviews of reviews (11). This design is the most appropriate method to synthesize evidence from a field with numerous systematic reviews (SRs) and heterogeneous findings, which is the case for this topic (12, 13). Reporting followed the PRIOR (Preferred Reporting Items for Overviews of Reviews) statement to ensure transparency and reproducibility (14).

### Search strategy

A comprehensive search strategy was developed with the assistance of a health sciences specialized librarian. The search was conducted in the following electronic databases from 2000 to April 2025: MEDLINE (via PubMed), Embase, Epistemonikos, Web of Science Core Collection, LILACS and Scopus. The strategy combined MeSH terms and keywords related to: (1) active video games (“active video gam*”, “exergam*”, “kinect”, “nintendo wii”, “pokémon go”, “dance dance revolution”); (2) physical activity (“physical activit*”, “exercise”, “motor activity”); and (3) systematic reviews (“systematic review”, “meta-analysis”). No language restrictions were applied.

### Selection criteria

To be included in this UR, studies had to meet specific criteria related to study type, population, intervention and outcomes. Exclusively SR with or without meta-analyses that synthesized quantitative evidence on the use of active video games and their impact on physical activity were included. These reviews had to include primary studies of experimental design (randomized controlled trials, controlled clinical trials, quasi-experimental studies with control group) or analytical observational studies (prospective or retrospective cohort studies, case-control studies, analytical cross-sectional studies). Reviews focused exclusively on functional rehabilitation, balance or coordination outcomes without physical activity measures were excluded, as well as conference abstracts, narrative reviews, scoping reviews, previous overviews, protocols without published results, and reviews that did not describe systematic and reproducible methods for search and study selection.

Also, reviews focused exclusively on functional rehabilitation, balance or coordination outcomes without physical activity measures were excluded. This distinction was made to maintain a clear focus on direct measures of physical activity volume and intensity (e.g., METs, MVPA, daily steps), rather than on measures of physical function or performance, thereby ensuring the homogeneity of the synthesized outcomes.

Regarding population, reviews that included participants of any age group (children, adolescents, adults, older adults) and in any health status were considered, both general population and groups with specific conditions (such as, for example, obesity). The intervention of interest comprised any type of active video game or exergame that required substantial physical movement for its control and progression, including, but not limited to games on commercial consoles (Nintendo Wii, Microsoft Xbox Kinect, PlayStation Move), virtual reality games with physical component, augmented reality mobile applications (such as Pokémon GO), and interactive dance or sports games. No restrictions were established for the comparator, accepting controls without intervention, waiting list, traditional exercise, sedentary video games or usual care.

The reviews had to report at least one outcome related to physical activity, whether measured objectively (through accelerometers, pedometers, indirect calorimetry, heart rate monitors) or subjectively (validated physical activity questionnaires, self-report). Outcomes of interest included, but were not limited to: general physical activity levels, time in MVPA, energy expenditure, number of steps, sedentary behavior, cardiorespiratory fitness, and adherence to physical activity recommendations. Reviews focused exclusively on functional rehabilitation, balance or coordination outcomes without physical activity measures were excluded, as well as narrative reviews, scoping reviews, previous overviews, protocols without published results, and reviews that did not describe systematic and reproducible methods for search and study selection.

### Selection process

All records identified in the searches were imported to Rayyan, a web application specifically designed to facilitate the selection process in SR. After eliminating duplicates automatically and manually, two independent reviewers evaluated titles and abstracts blindly using Rayyan's functionalities that allow hiding the other reviewer's decisions. Conflicts were resolved through discussion after completing the initial evaluation, and when consensus was not reached, a third reviewer made the final decision. Articles considered potentially eligible or those with insufficient information in the abstract were obtained in full text and were independently evaluated by the same reviewers following the same process. Specific reasons for exclusion in the full-text phase were documented in Rayyan and were reported following the PRISMA 2020 flow diagram (15).

### Data extraction

A standardized data extraction form was developed in Microsoft Excel, which was piloted with five randomly selected reviews and refined as needed. Two reviewers independently extracted the following data: (1) bibliographic and methodological characteristics of the review (authors, year of publication, main objective, number and type of databases consulted, search period, number of studies included, total participants, tool used for quality assessment); (2) characteristics of included primary studies (study designs, sample size ranges, follow-up duration); (3) population characteristics (age groups, sex distribution, special health conditions, geographical context); (4) intervention details (specific types of active video games, platforms used, intervention duration, frequency and intensity of sessions, implementation context); (5) physical activity measurement methods (objective and subjective instruments used); (6) main and secondary outcomes related to physical activity, including effect measures when available (mean differences, effect sizes, odds ratios with their respective confidence intervals); (7) statistical heterogeneity measures when meta-analysis was performed; and (8) main conclusions and recommendations from the authors. Extracted data were cross-verified and discrepancies were resolved by consulting the original articles again.

### Methodological quality assessment

The methodological quality of included SR was assessed using AMSTAR 2 (A MeaSurement Tool to Assess systematic Reviews 2), a validated 16-item tool that provides a comprehensive evaluation of SR of randomized and non-randomized studies (16). Two reviewers independently applied AMSTAR 2 to each included review, evaluating critical domains (registered protocol, adequate search, justification of exclusions, risk of bias assessment, appropriate meta-analytical methods, consideration of risk of bias in interpretation, publication bias assessment) and non-critical domains. Overall quality was categorized as high, moderate, low or critically low according to AMSTAR 2 criteria. For reviews that included predominantly non-randomized studies, ROBIS (Risk of Bias in Systematic Reviews) was complementarily applied to assess risk of bias in four domains: eligibility, identification and selection of studies, data collection and evaluation, and synthesis and findings (17).

### Data synthesis

Given the anticipated heterogeneity in populations, interventions and outcome measures, a structured narrative synthesis was performed following the framework of the Cochrane Consumers and Communication Review Group (18). Findings were organized by: (1) population group (children, adolescents, adults, older adults, special populations); (2) type of AVG (commercial consoles, virtual reality, mobile applications); (3) intervention context (school, home, community, clinical); and (4) type of physical activity outcome. When multiple reviews addressed the same research question, the most recent and highest methodological quality were prioritized. The degree of overlap between reviews was calculated using the corrected covered area (CCA) according to Pieper et al.'s methodology (19). Reported effect sizes were converted to a common metric (Hedges' g) when possible to facilitate comparisons. To clarify our decision rule, a *de novo* meta-analysis (i.e., a meta-meta-analysis) was not performed for two main reasons. First, the included reviews exhibited extreme clinical and methodological heterogeneity, which would make a quantitative synthesis inappropriate. Second, the anticipated overlap of primary studies across reviews created a significant risk of double-counting evidence, violating the statistical assumption of independence and leading to invalid pooled estimates. Therefore, our primary approach was a structured narrative synthesis. We extracted and reported pooled quantitative results from the original systematic reviews only when a review conducted a meta-analysis on a primary outcome of interest (e.g., overall physical activity, fitness in a specific subgroup) that was central to illustrating this UR's key findings.

### Certainty of evidence assessment

The certainty of the body of evidence for each outcome was assessed using an adaptation of the GRADE (Grading of Recommendations Assessment, Development and Evaluation) approach for URs, considering: methodological quality of included reviews, consistency of findings between reviews, precision of estimates, evidence of publication bias, and applicability of evidence (20). Certainty was classified as high, moderate, low or very low for each key outcome.

## Results

### Study selection

**Figure 1:**
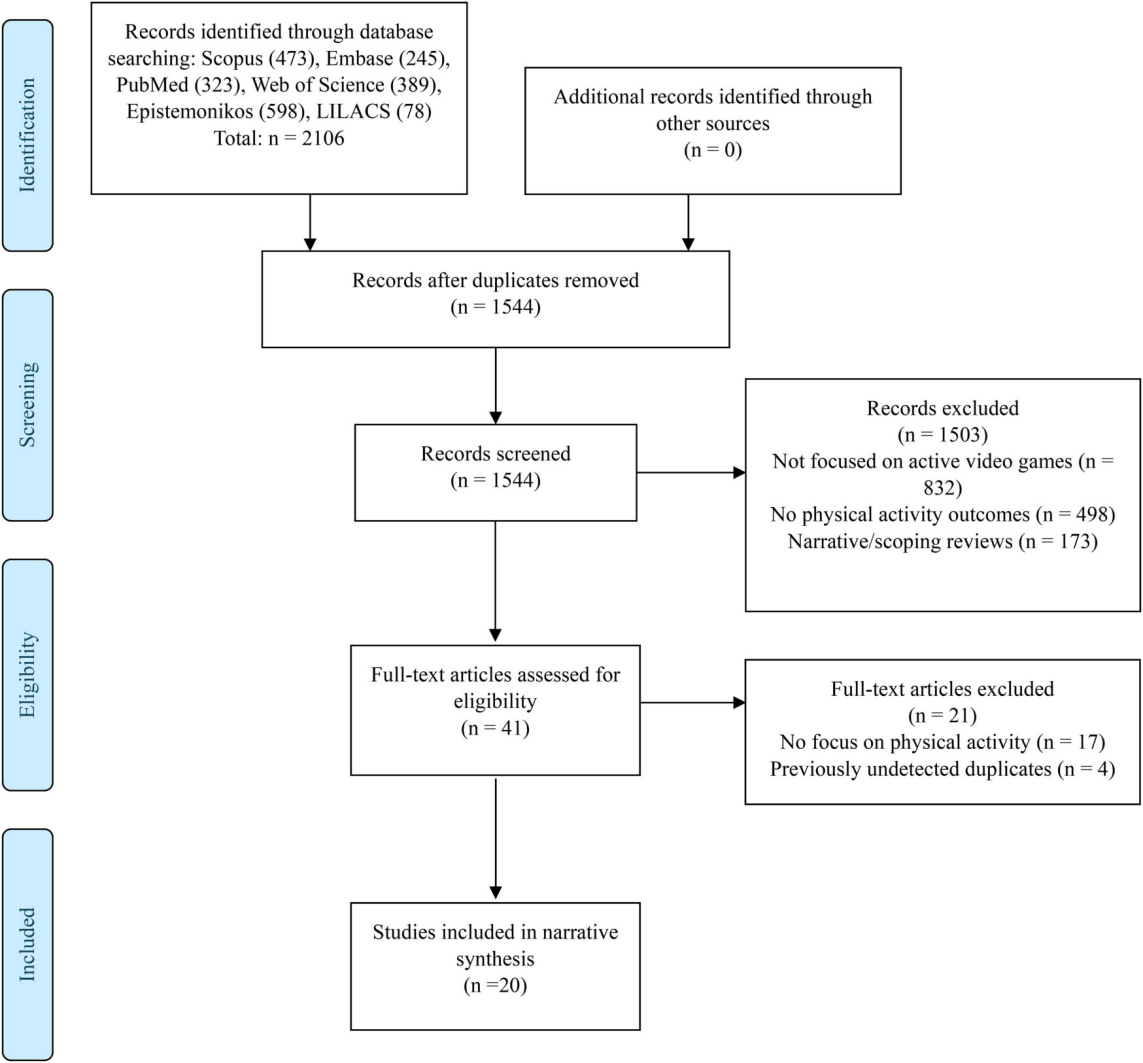
Flowchart of study selection.

The systematic search in six databases identified 2,106 records, with no additional records from other sources. After removing 562 duplicates, 1,544 records were screened by title and abstract. Of these, 1,503 were excluded mainly for not focusing on active video games (*n* = 832), lacking physical activity outcomes (*n* = 498) or being narrative/scoping reviews (*n* = 173). Forty-one articles were evaluated at full text, excluding 21 mainly for lack of focus on physical activity (*n* = 17) and duplicates not previously detected (*n* = 4). Finally, 20 SR met the eligibility criteria and were included in the narrative synthesis (8, 21–39) (Figure 1).

### Characteristics of included reviews

The 20 included SR were published between 2013 and 2025, with a marked acceleration in scientific production in this field observed during the last five years, with 12 reviews published between 2020 and 2025 (Table 1). This temporal trend reflects the growing interest in technological interventions for physical activity promotion, particularly after the COVID-19 pandemic. The temporal scope of bibliographic searches showed considerable variability, from reviews that searched literature from the beginnings of databases in 1996 (23) to those that deliberately limited their search to the last five years to capture the most recent technologies (30). This variability in search periods reflects different research objectives, with some reviews seeking to understand the historical evolution of the field and others focusing on the most current evidence.

**Table 1:** Characteristics of selected SR.

Author/Year	Main objective	Search (databases/period)	Included studies (*n*, designs)	Participants (*n*, characteristics)	AVG interventions	Physical activity measures	Main findings (effect sizes)	Methodological quality	Key conclusions
Norris et al. (2016) (22)	Evaluate effects of AVG in schools on physical activity and health	5 databases/April-May 2015	22 reports (7 RCT, 2 CT, 8 repeated measures, 5 pre-post)	3,728 (5-15 years, 46.2% girls)	Nintendo Wii, PlayStation 2, Xbox 360, DDR	MVPA, steps, energy expenditure (accelerometers, pedometers)	9/14 studies showed greater PA in AVG sessions vs. controls. Mixed results	Low (16 studies), Moderate (6 studies)	Insufficient evidence to recommend AVG as effective interventions in schools
Peng et al. (2013) (8)	Evaluate AVG interventions to increase PA and quantify intensity	5 databases/Until March 2011	41 (28 laboratory, 13 interventions, 8 RCT)	3,371 total (children and adults)	Commercial AVG (Wii, Xbox, DDR, GameBike)	METs, VO₂, caloric expenditure, HR	Laboratory: AVG = light-moderate PA. Interventions: only 3/13 significantly increased PA	22–100 points (mea*n* = 68)	AVG provide light-moderate PA, but little support for long-term efficacy
Höchsmann et al. (2015) (21)	Evaluate effectiveness of exergaming in individuals with overweight and type 2 diabetes	5 databases/Beginning-March 2015	11 studies (14 publications)	268 overweight adults (BMI ≥25), 19.7–70.7 years	Nintendo Wii, PlayStation, Xbox Kinect, GameBike, DDR	VO₂, energy expenditure, HR, METs	VO₂ increase (49%–316%), energy expenditure (1.5–5x), HR (39%–102%); intensity mostly light	1 low risk, 3 high, 7 unclear	Exergames can increase PA in overweight people, but inconsistent results
Khamzina et al. (2019) (26)	Quantify relationship between Pokémon Go and PA	8 databases/July 2016–Oct 2018	17 (16 observational, 1 pre-post)	33,108 (mostly adults, ∼70% men)	Pokémon Go (AR mobile app)	Steps/day, PA time, distance (accelerometers, apps)	Increase 1,446 steps/day (95% CI: 953–1,939), *I*^2^ = 81%	Low (16), Moderate (6)	Pokémon Go associated with modest but significant increase in daily steps
Street et al. (2017) (24)	Explore use of exergaming to promote PA and health in adults	5 databases/Beginning-Nov 2016	9 studies	NR total, adults ≥18 years	Nintendo Wii, Xbox, PlayStation, GameBike, DDR	Changes in PA, accelerometers	Short-term PA increase; 1.5–6 METs; 30% higher adherence vs. traditional exercise	Poor to good (Wesley Protocol)	Exergaming effective short-term if intensity and duration specified
Zheng et al. (2019) (27)	Evaluate exergames in frail/pre-frail elderly and their physical impact	5 databases/Until January 2019	7 studies	243 frail older adults, 69.5–85.47 years	Xbox 360, Nintendo Wii, Fovea Interactive, Personics, FRED	Strength, balance, mobility, gait	Balance and mobility improvement; high adherence (100% in one study)	Acceptable, variable risk	Exergames improve balance/mobility; trend to increase strength
Williams et al. (2020) (28)	Explore whether AVG improve PA in adolescents	6 databases/2007–2019	6 RCTs	20–105 per study (12–19 years)	DDR, Kinect, GameBike	Accelerometers, Fitbits, pedometers	5/6 studies showed improvement in PA levels	Cochrane tool	AVG appear to be safe and valuable means to promote PA in adolescents
Gao et al., 2020 (29)	Synthesize effects of AVG on body composition and PA in youth	9 databases/2000-Dec 2019	18 RCTs	6–19 years (majority)	Wii, Xbox Kinect, DDR	Objective PA (accelerometers)	Positive effects in overweight/obesity; neutral effects in normal weight (10/16 studies)	High quality (13/18)	AVG promising for overweight/obese youth, less clear for normal weight
Ramírez-Granizo et al. (2020) (30)	Review relationship between exergames and PA	WOS/2016–2020	8 (longitudinal and experimental)	1,004 (6–16 years)	Kinect, Wii Fit, Nintendo	Accelerometers, pedometers	Favor motivation toward exercise with adequate parameters	PRISMA guidelines	Exergames can complement physical education or substitute sedentarism
Lee et al., (2021) (31)	Examine effects of Pokémon Go on PA and wellbeing	6 databases/July 2016–April 2021	36 (33 observational, 3 experimental)	38,724 (5≥60 years)	Pokémon Go	Steps/day, PA time (pedometers, apps)	Greater PA in players vs. non-players	Weak (23), Moderate (11), Strong (2)	Pokémon Go promotes significant improvements in walking behavior
Moller et al. (2023) (32)	Meta-analysis of AVG interventions on PA	6 databases/Until Dec 2020	25 RCTs (19 in meta-analysis)	2,888 (mean 18.6 years)	Wii (44%), Kinect (36%), PlayStation (16%)	Post-intervention PA	Hedges g = 0.525 (95% CI: 0.322–0.728), *I*^2^ = 87.7%	Low GRADE	AVG promising tool but with high variability in quality and design
Chen et al. (2023) (33)	Compare exergames vs. conventional exercises in older adults	6 databases/Until Feb 2023	12 RCTs (10 meta-analysis)	919 older adults ≥60 years	Wii (4), Kinect (5), Tymo (1), GRAIL (1)	30-s chair stand, gait, Berg balance	No significant difference between exergames and conventional exercises	Acceptable, high performance bias	No significant difference between exergames and conventional exercises
Liang et al. (2023) (34)	Impact of Pokémon Go on PA and wellbeing in children/adolescents	4 databases/Until March 2023	10 (various designs)	13–944 (<18 years)	Pokémon Go	PA and psychosocial wellbeing	Positive impact on PA; mixed results on wellbeing	Moderate-low GRADE	Pokémon Go positively affects PA, psychosocial impact requires more research
Zhao et al. (2024) (36)	Effects of AVG on PA in overweight/obese university students	5 databases/Until 2022	8 RCTs	234 university students	Wii, Xbox, PlayStation	%VO2max, METs, MVPA	5/6 studies: AVG achieve moderate intensity; 1 study: vigorous intensity	PEDro 4–7 points	AVG viable intervention to increase PA in overweight university students
Deng et al. (2024) (37)	Meta-analysis AVG on physical fitness of older adults	7 databases/Until January 2024	24 RCTs	1,428 (≥60 years, 72% women)	Nintendo Wii mainly	Strength, cardiorespiratory fitness	Muscle strength: ES = 0.64–0.68 (*p* < 0.05); Cardio: ES = 0.79 (*p* < 0.001)	Moderate-high PEDro	AVG effective for strength and cardiorespiratory fitness in older adults
Liu et al. (2024) (38)	Effectiveness of serious games on body composition, PA and diet	6 databases/Until April 2023	20 RCTs	4,221 children/adolescents	Educational serious games	Accelerometers	PA: SMD = 0.61 (95% CI: 0.04–1.19); Body composition: SMD = -0.26 (NS)	RoB 2	Serious games show potential, especially for PA
Spring et al. (2025) (39)	Effects of exergaming on BMI and PA in children/adolescents with obesity	2 databases/Period NR	12 studies	29–445 per study (5–19 years)	Video game consoles	BMI, BMI-z, body composition, PA	Promising effects on MVPA; subtle to moderate effect on BMI	Covidence and Cochrane	Exergames potential as adjunct tools in pediatric obesity
Lamas et al. (2023) (35)	Influence of serious games on healthy diet and PA	4 databases/2003–2021	26 (15 RCT, 11 quasi-exp)	8,844 (3–18 years)	Serious games, applications	Questionnaires	Majority of nutritional interventions effective; PA only during game	NR	Serious games effective for nutritional education
Pakarinen et al. (2016) (23)	Health game interventions on PA self-efficacy in children	5 databases/1996–2016	5 (4 RCT, 1 quasi-exp)	NR (<18 years)	Wii, DDR, mobile apps	Self-efficacy scales	3 studies with significant increases in self-efficacy	Cochrane medium risk	Active games can be used as independent interventions
Taylor et al. (2018) (25)	Meta-analysis AVG on physical performance of older adults	3 databases/Until April 2015	18 RCTs	765 (>65 years)	Nintendo Wii (11 studies)	Mobility, balance (TUG, BBS)	Berg Balance: MD = 4.33 (95% CI: 2.93–5.73) vs. conventional exercise	Variable Cochrane	AVG improve mobility and balance in older adults

PA, physical activity; AVG, active video games; AR, augmented reality; BBS, Berg balance scale; CI, confidence interval; CT, controlled trial; DDR, dance dance revolution; ES, effect size; HR, heart rate; MD, mean difference; METs, metabolic equivalents; MVPA, moderate to vigorous physical activity; NR, not reported; NS, not significant; RCT, randomized controlled trial; SMD, standardized mean difference; TUG, timed up and go.

The rigor of bibliographic searches varied notably between reviews. The number of databases consulted ranged between 2 Gao et al. (29) and 9 Spring et al. (39), with a median of 5 databases per review. The most frequently used databases were PubMed/MEDLINE (consulted by all 20 reviews, 100%), Web of Science (18/20, 90%), SPORTDiscus (14/20, 70%), Cochrane Library (13/20, 65%), and EMBASE (12/20, 60%). The inclusion of specialized databases in sports and exercise sciences such as SPORTDiscus was more common in reviews published after 2018, suggesting a growing recognition of the interdisciplinary nature of the field. The comprehensiveness of search strategies also showed significant variability, with only 11 reviews reporting systematic search in grey literature through repositories such as OpenGrey or ProQuest Dissertations, and only 9 reviews documenting manual search in the references of included articles or consultation with field experts.

### Characteristics of primary studies

The aggregate analysis revealed that the 20 SR included a total of 418 unique primary studies, although the calculation of the corrected covered area (CCA) indicated a high overlap of 12.3% between reviews. This overlap was particularly notable between reviews that evaluated interventions in older adults, where studies on Nintendo Wii appeared in multiple reviews. The number of studies included per review showed a wide distribution, from 5 studies in the most selective review Pakarinen et al. (23) to 41 studies in the most inclusive Peng et al. (8), with a median of 12 studies and an interquartile range of 8–20 studies.

The distribution of research designs revealed interesting patterns about the methodological evolution of the field. Twelve reviews (60%) included exclusively randomized controlled trials, reflecting a preference for maximum experimental rigor. Six reviews (30%) adopted a mixed approach, combining RCTs with observational, quasi-experimental or pre-post designs, recognizing the value of different methodological approaches. Finally, 3 reviews were based predominantly on observational studies, specifically the reviews on Pokémon Go (26, 31, 34), where the nature of the phenomenon—spontaneous mass adoption of a mobile application—lent itself more to naturalistic than experimental studies. In Lee's review (31), for example, 33 of the 36 included studies were observational, leveraging natural data generated by millions of users. This distribution reflects both the maturation of the field toward more rigorous designs and the pragmatic recognition that certain phenomena require diverse methodological approaches.

The aggregate sample size showed extreme variability between reviews, reflecting the diversity of methodological approaches and populations studied. Total sample sizes varied from 234 participants in Zhao's focused review (36) on overweight university students, to 38,724 participants in Lee's review (31) on Pokémon Go. This difference of more than 165 times in sample size illustrates the fundamental heterogeneity in the field, where small but rigorous experimental studies coexist with large population observational studies. The duration of interventions in primary studies also showed notable variability, from single 60-min experimental sessions designed to evaluate acute physiological responses, to intervention programs that extended over complete academic periods. The longest intervention identified was reported by Norris et al. (22), with a program that was implemented for two complete academic years in the school context.

### Populations studied

The analysis of populations included in the reviews revealed comprehensive coverage of the age spectrum, although with unequal distributions that reflect research priorities and practical feasibility. Nine reviews focused exclusively on pediatric and adolescent population, including the works of Norris et al. (22), Pakarinen et al. (23), Williams et al. (28), Gao et al. (29), Ramírez-Granizo et al. (30), Liang et al. (34), Lamas et al. (35), Liu et al. (38), Spring et al. (39). This concentration on young population reflects both the concern about growing levels of physical inactivity in childhood and this age group's greater familiarity with video game technologies.

Four reviews focused specifically on older adults, represented by the works of Taylor et al. (25), Zheng et al. (27), Chen et al. (33), Deng et al. (37). This substantial interest in geriatric population reflects the recognition of AVG potential to overcome traditional barriers to exercise in older adults, such as mobility problems, fear of falls, and social isolation. Two reviews (10%) focused on adults with specific health conditions: Höchsmann et al. (21) in adults with overweight and type 2 diabetes, and Zhao et al. (36) in university students with overweight or obesity. The remaining four reviews adopted a more inclusive approach, encompassing multiple age groups in their analyses.

Sex distribution was reported inconsistently across reviews, with only 12 of the 20 reviews (54.5%) providing disaggregated data by this variable. Among those that reported this data, interesting patterns were observed. Norris et al. (22) reported a relatively balanced distribution with 46.2% female participation in school studies. In contrast, reviews in older adults showed marked female predominance, with Deng et al. (37) reporting 72% women participants and Chen et al. (33) finding similar proportions. On the other hand, reviews on Pokémon Go showed male predominance, with Khamzina et al. (26) reporting approximately 70% male participants, possibly reflecting gender patterns in mobile video game use.

The geographical distribution of primary studies revealed an overwhelming concentration in high-income countries, raising serious questions about the global generalizability of findings. The United States emerged as the dominant country in AVG research, appearing in all reviews that reported geographical data. The proportion of US studies varied from 48% in Moller's global review (32) to 58% in the reviews by Lee et al. (31) and Lamas et al. (35). Other frequently represented high-income countries included United Kingdom (present in 13 reviews), Canada (11 reviews), Australia (9 reviews), and several European countries including Germany, France, Italy and the Netherlands.

The representation of middle and low-income countries was notably scarce, limited to isolated mentions in few reviews. The only Latin American countries represented were Brazil, mentioned in Zhao's review (36), Mexico in Lamas et al. (35), and Peru in Liang et al. (34). From Asia, in addition to high-income countries like Japan and South Korea, only Indonesia appeared in two reviews. Africa was completely absent from all analyzed reviews. This biased geographical distribution has important implications, as physical activity patterns, technology access, and cultural factors related to gaming may differ substantially between socioeconomic contexts.

### Types of active video game interventions

The analysis of technological platforms used revealed both the temporal evolution of the field and persistent preferences for certain technologies. Nintendo Wii emerged as the most studied platform, appearing in 16 of the 20 reviews. This dominance reflects several factors: it was one of the first commercially successful consoles to incorporate motion control, its intuitive interface made it accessible to users unfamiliar with traditional video games, and its relatively accessible price facilitated its adoption in research contexts. The most frequently used specific Wii games included Wii Sports (especially bowling and tennis), Wii Fit (with its exercise and balance routines), and Just Dance.

Microsoft Xbox Kinect represented the second most common platform, present in 11 reviews. Introduced in 2010, Kinect offered technological advantages over Wii, including full-body detection without need for manual controllers and ability to track multiple players simultaneously. The most used Kinect games in interventions included Kinect Sports, Kinect Adventures, and Your Shape: Fitness Evolved. Dance Dance Revolution (DDR), despite being older technology, maintained a significant presence appearing in 8 reviews, particularly in studies with adolescents where its social and musical component was especially attractive.

Pokémon Go represented a unique phenomenon in AVG literature, being the exclusive focus of three complete reviews, those of Khamzina et al. (26), Liang et al. (34), and Lee et al. (31) which together analyzed 63 primary studies. As the first augmented reality application to achieve massive global adoption, Pokémon Go differed fundamentally from traditional AVG by requiring physical displacement in the real world to progress in the game.

The frequency and intensity of use showed diverse patterns that reflected different intervention philosophies and implementation contexts. At the minimal extreme, several studies included in Peng's review (8) used single laboratory sessions of 15–60 min, designed primarily to evaluate acute physiological responses such as energy expenditure and heart rate. At the opposite extreme, school interventions such as those reviewed by Norris et al. (22) implemented programs of up to 5 weekly sessions during complete academic periods. The modal session duration was 30 min, although with ranges from 10 min for specific high-intensity games to 60 min for sessions that included multiple games or additional educational components.

The implementation context emerged as a crucial factor that influenced both the design and outcomes of interventions. School environments, extensively analyzed by Norris et al. (22) and present in 9 additional reviews, offered advantages of access to large populations and possibility of curricular integration, but faced challenges of limited time, shared resources, and need for supervision. Home environments, evaluated in reviews such as Williams et al. (28) and Street et al. (24), allowed greater flexibility and potential for sustained use, but suffered from lower experimental control and higher dropout rates. Research laboratories, prominent in the reviews by Peng et al. (8) and Höchsmann (21), provided maximum experimental control and precise measurements, but questionable ecological validity. Community and clinical environments, particularly relevant in older adult reviews such as Taylor et al. (25) and Zheng et al. (27), offered a balance between professional supervision and naturalistic context.

### Physical activity measures

Regarding heterogeneity, this variability was not merely technical but reflected fundamentally different conceptualizations of what constitutes “effective physical activity” in the context of AVG. Objective measures dominated the literature, with accelerometers being the most common tool, used in 16 of the 20 reviews. However, even within this apparently homogeneous category, variability was substantial: different brands and models of accelerometers (ActiGraph, Actical, RT3), different placement locations (hip, wrist, ankle), different sampling epochs (1 s–60 s), and crucially, different algorithms and cut points for classifying activity intensities.

Pedometers, used in 9 reviews, offered simplicity and low cost but significant limitations by capturing only ambulatory movement, potentially underestimating activity during games that involved primarily upper extremity movements. Heart rate monitors, present in 8 reviews, provided continuous measures of physiological intensity but faced challenges of individual calibration and the influence of factors unrelated to exercise such as stress or emotional excitement during gameplay. Indirect calorimetry, gold standard for energy expenditure measurement, appeared mainly in laboratory studies included in 4 reviews, limited by its cost, technical complexity, and restriction to controlled environments.

The specific variables measured showed a diversity that extremely complicated comparisons between studies. Time in MVPA was reported by 11 reviews but with variable definitions: some studies used the traditional threshold of 3 METs, others 4 METs, and some applied age-specific thresholds. METs were reported by 7 reviews, but some as absolute values and others as percentages of age-estimated maximum. Energy expenditure appeared in 6 reviews but expressed alternatively as total kcal, kcal/minute, kcal/kg/hour, or kJ, requiring complex conversions for comparison. Daily steps, prominent in Pokémon Go reviews and some pedometer interventions, varied in whether they included all steps of the day or only those attributable to the intervention.

Subjective measures, present in 12 reviews, added another layer of complexity. The International Physical Activity Questionnaire (IPAQ) appeared in various forms (short, long, modified) with variable recall periods. Instruments specific for pediatric populations such as the Physical Activity Questionnaire for Children (PAQ-C) and the System for Observing Play and Active Recreation in Kids (SOPARK) had variable psychometric properties according to cultural context. The diversity was such that Ramírez-Granizo et al. (30) explicitly noted that the “very disparate results” were largely due to “the wide variety of contexts, instruments used, duration and methodologies,” an observation that resonated across multiple reviews.

### Effectiveness of interventions

The findings on AVG effectiveness for promoting physical activity presented a complex panorama characterized by substantial heterogeneity but with identifiable patterns according to population and context. The most comprehensive meta-analysis, conducted by Moller et al. (32) and including 19 studies with 2,888 participants, reported a moderate general positive effect (Hedges g = 0.525, 95% CI: 0.322–0.728). This effect size, although statistically significant, must be interpreted in the context of extremely high heterogeneity (*I*^2^ = 87.7%), indicating that the true effect probably varies substantially according to moderating factors. To contextualize, this effect is comparable to traditional school physical activity interventions but smaller than supervised structured exercise programs.

The interpretation of these findings varied considerably between review authors. Norris et al. (22), adopting more strict criteria, concluded that there is “insufficient evidence to recommend AVG as effective health interventions in schools” after finding that only 9 of 14 studies (64.3%) showed greater physical activity in AVG sessions compared to controls. This more conservative conclusion reflected not only the mixed results but also concerns about the methodological quality of primary studies, the lack of physical activity measures outside the school context, and the absence of evaluation of possible compensatory effects.

The analysis by subpopulations revealed more consistent patterns. For older adults, the evidence was particularly robust. Deng et al. (37), analyzing 24 randomized controlled trials with 1,428 participants, found significant and clinically relevant effects: effect sizes for muscle strength of ES = 0.64–0.68 (*p* < 0.05) and for cardiorespiratory fitness of ES = 0.79 (*p* < 0.001). These effects were not only statistically significant but comparable to traditional exercise programs in this population. The complementary review by Chen et al. (33), while not finding significant differences between exergames and conventional exercise, noted the importance that AVG can achieve similar benefits to traditional exercise while potentially offering greater adherence and enjoyment.

In the case of pediatric populations with overweight or obesity, studies such as Gao et al. (29) identified an important differential pattern analyzing 18 RCTs, as while AVG showed consistent positive effects in overweight/obese youth, in normal-weight youth more than half of the studies (*n* = 10) demonstrated neutral effects. Spring et al. (39), focusing specifically on pediatric obesity, concluded that exergames have “potential as adjunct tools in pediatric obesity treatment,” although noting “subtle to moderate” effects on BMI.

Specifically, in the case of reviews focused on Pokémon Go, we can highlight the study by Khamzina et al. (26), synthesizing 17 studies with more than 33,000 participants, reported an average increase of 1,446 daily steps (95% CI: 953–1,939). Although this increase represents approximately 14% of the daily recommendation of 10,000 steps, noting that this was completely voluntary and without formal intervention. Lee et al. (31), with an even larger sample of 38,724 participants, confirmed these findings and added that “players had significantly greater physical activity than non-players in terms of daily steps and number of days dedicated to moderate physical activity.” However, both reviews noted high heterogeneity (*I*^2^ = 81%) and recognized the limitations of predominantly observational designs.

The intensity achieved during gameplay provided one of the most consistent findings. Multiple reviews converged on AVG typically achieving light to moderate intensity (3–6 METs). Peng et al. (8) established early that “all laboratory studies showed that AVG provide light to moderate intensity physical activity.” This observation was confirmed by subsequent reviews, with Höchsmann et al. (21) reporting increases in VO₂ of 49%–316% over rest and Street et al. (24) confirming levels of 1.5–6 METs. Importantly, certain types of games consistently achieved higher intensities: dance, boxing games, and those requiring full-body movements frequently reached the vigorous activity threshold (>6 METs).

### Duration and sustainability of effects

The question of sustainability emerged as one of the most critical and consistent limitations across reviews. Peng et al. (8) identified this concern early, reporting that only 3 of 13 interventions showed significant sustained increases in physical activity beyond the active intervention period. This pessimistic observation was confirmed and elaborated by subsequent reviews, establishing a concerning pattern of temporally limited effects.

Liu et al. (38) provided quantitative evidence on the importance of intervention duration, finding that longer interventions (>3 months) showed greater effects on body composition (SMD = −0.40, 95% CI: −1.13 to 0.33) compared to shorter interventions (≤3 months, SMD = −0.02, 95% CI: −0.33 to 0.30), although the difference did not reach statistical significance (*p* = 0.24). This trend was supported by Deng et al. (37), who reported more definitive findings: “the beneficial effects of AVGs were greater after ≥12 weeks vs. < 12 weeks for cardiorespiratory fitness (ES = 1.04 vs. 0.29, *p* = 0.028).” These findings suggest that, similar to traditional exercise interventions, a minimum exposure period is required to achieve significant physiological adaptations.

The phenomenon of diminishing interest and participation over time was consistently documented. Street et al. (24) explicitly noted “decrease in participation over time” as a key finding, while Lamas et al. (35) offered the most direct observation: “games directed at physical activity were not effective after the game, only during.” This temporal limitation suggests that AVG may be more effective as a tool to initiate physical activity than to maintain it long-term, raising important questions about their value as sustainable public health intervention.

The case of Pokémon Go provided a natural example of this phenomenon at population scale. While the initial launch period in July 2016 generated dramatic increases in physical activity, multiple longitudinal studies included in the reviews documented gradual decreases. The typical curve showed peak activity in the first 2–4 weeks, followed by gradual decline, although many users maintained activity levels superior to baseline even after months. This “novelty-decline” pattern is not unique to AVG, but poses particular challenges for interventions that depend on user engagement and interaction with technology.

### Methodological quality of reviews

The systematic evaluation of methodological quality through AMSTAR 2 revealed limitations in the reported findings (Supplementary Material S2). Of all evaluated reviews, only 3 achieved high methodological quality rating: Moller et al. (32), Deng et al. (37), and Chen et al. (33). These exemplary reviews met all or almost all 16 AMSTAR 2 criteria, including the seven critical domains.

Six reviews obtained moderate quality rating, meeting most criteria but with deficiencies in one or two critical domains. Moderate quality reviews included influential works such as those by Höchsmann et al. (21), Khamzina et al. (26), and Gao et al. (29). Eight reviews were rated as low quality, typically failing multiple critical domains but maintaining some elements of methodological rigor. Concerningly, three reviews received critically low rating, including Ramírez-Granizo et al. (30), Spring et al. (39), and Lamas et al. (35), indicating methodological flaws so severe that the results should not be considered reliable.

The complementary evaluation with ROBIS for the eight reviews that included predominantly non-randomized studies revealed additional concerns about risk of bias. Five of these eight reviews (62.5%) showed high risk of bias, mainly in the synthesis and findings domains. The most common deficiencies included inappropriate synthesis of heterogeneous designs without adequate stratification and lack of consideration of risk of bias in results interpretation. Only three reviews (27, 31, 34) showed unclear rather than high risk, mainly due to better methodological reporting and appropriate consideration of observational design limitations (Supplementary Table S3).

The absence of *a priori* registered protocol was the most common deficiency, present in only 6 of the 20 reviews. This lack of pre-registration increases the risk of selective reporting bias and *post-hoc* decisions on inclusion criteria or analysis. The provision of a list of excluded studies with justifications, fundamental for transparency, was adequate in only 4 reviews. The evaluation of publication bias, critical for interpreting the validity of quantitative syntheses, was appropriately performed in only 11 reviews, and frequently limited to visual inspection of funnel plots without formal statistical tests.

### Heterogeneity and moderating factors

Statistical heterogeneity emerged as a universal finding in reviews that performed meta-analysis, with *I*^2^ values consistently superior to 75%, indicating substantial to considerable heterogeneity according to Cochrane criteria. Moller et al. (32) reported *I*^2^ = 87.7% for their main analysis, while Liu et al. (38) found even more extreme heterogeneity for body composition (*I*^2^ = 83%) and physical activity (*I*^2^ = 92%). This heterogeneity was not merely a statistical problem but reflected genuine variability in effects according to multiple moderating factors.

Age emerged as the most consistently identified moderator. Liu et al. (38) provided clear quantitative evidence: effects on body composition were substantially greater in children <14 years (SMD = −0.40, 95% CI: −1.13 to 0.33) compared to adolescents ≥14 years (SMD = −0.01, 95% CI: −0.17 to 0.15). This pattern may reflect greater behavioral plasticity in younger children, less self-consciousness during gameplay, or simply greater enthusiasm for gaming formats. Paradoxically, older adults also showed superior responses, suggesting a U-shaped relationship with age.

The type of game and technological platform emerged as another critical moderator. Peng et al. (8) established the fundamental principle: “Full-body or lower limb AVGs produce greater energy expenditure than upper limb ones.” This intuitive but important finding was quantified by Moller et al. (32), who found graduated effects according to the level of body involvement: simple step devices (Hedges g = 0.303, 95% CI: 0.110–0.496), combination of manual devices and body detection (Hedges g = 0.512, 95% CI: 0.288–0.736), and full-body systems (Hedges g = 0.694, 95% CI: 0.350–1.039).

### Adverse events and safety

Only 6 of the 20 reviews reported systematic search and synthesis of safety data, and even among these, reporting was frequently superficial. This negligence is particularly problematic given that AVG are frequently promoted for vulnerable populations such as older adults at risk of falls or children with medical conditions.

Among reviews that did evaluate safety, findings were generally limited. Taylor et al. (25), focusing on older adults, did not identify serious adverse events in 18 controlled trials, although noted occasional reports of mild muscle pain and fatigue. Zheng et al. (27) reported high acceptability and absence of serious adverse events in frail older adults, although with the caveat that studies may have excluded higher-risk participants. For Pokémon Go, safety concerns were qualitatively different, focusing on distraction and accident risks during displacement. Khamzina et al. (26), Liang et al. (34) and Lee et al. (31) mentioned media reports of game-related accidents, although none provided systematic data on incidence or severity.

### Certainty of evidence assessment

The evaluation of evidence certainty through the GRADE system revealed marked variability in the confidence we can have in the different findings of this UR, reflecting both the inherent heterogeneity of the field and the identified methodological limitations (Table 2). This evaluation process systematically considered domains of risk of bias, inconsistency, indirect evidence, imprecision and publication bias, as well as factors that could increase confidence such as large effect sizes, dose-response gradients, and situations where plausible biases would reduce a demonstrated effect.

**Table 2:** GRADE evaluation of evidence certainty for main outcomes.

Outcome	No. of reviews (No. primary studies)*	No. participants**	Evidence certainty	Factors modifying certainty	Summary of findings
General physical activity levels	15 (230)	19,383	⊕⊕⊕⊝ MODERATE	Serious inconsistency (−1)^a^ Methodological limitations (−1)^b^ Moderate effect size (+1)^c^	AVG probably increase physical activity moderately (Hedges g = 0.525, 95% CI: 0.322–0.728)
Daily steps	3 (54)	72,556	⊕⊕⊝⊝ LOW	Serious inconsistency (−1)^d^ Study design (−1)^e^	AVG might increase ∼1,446 steps/day, although evidence is uncertain due to high variability
Time in MVPA	10 (134)	10,196	⊕⊕⊝⊝ LOW	Serious inconsistency (−1)^f^ Imprecision (−1)^g^ Risk of bias (−1)^h^ Direct objective measurement (+1)^i^	AVG might increase time in MVPA, although effects are inconsistent between studies
Intensity during gameplay (METs)	7 (93)	4,904	⊕⊕⊕⊝ MODERATE	Methodological limitations (−1)^j^ Consistency between studies (+1)^k^ Direct objective measurement (+1)^l^	AVG consistently achieve light-moderate intensity (3–6 METs) during sessions
Physical fitness in older adults	5 (80)	3,355	⊕⊕⊕⊕ HIGH	No serious degradation Large effect size (+1)^m^ High consistency (+1)^n^	AVG significantly improve strength (ES = 0.64–0.68) and cardiorespiratory fitness (ES = 0.79)
Physical activity in people with overweight/obesity***	6 (68)	2,279	⊕⊕⊕⊝ MODERATE	Imprecision (−1)^o^ Clinically relevant effect (+1)^p^ Objective measurement (+1)^g^	AVG are probably effective in this population, with consistent benefits
Sustainability of effects (>6 months)	7 (58)	4,629	⊕⊝⊝⊝ VERY LOW	Very serious inconsistency (−2)ʳSerious imprecision (−1)^s^ Indirect evidence (−1)^t^	Very uncertain evidence: only 3/13 interventions showed sustained effects
Safety/Adverse events	6 (74)	5,502	⊕⊝⊝⊝ VERY LOW	Very limited reporting (−2)^u^ Absence of standardized protocols (−1)^v^	Fragmentary data prevent establishing reliable risk profile. No serious events reported

GRADE rating system: ⊕⊕⊕⊕ High certainty; ⊕⊕⊕⊝ Moderate certainty; ⊕⊕⊝⊝ Low certainty; ⊕⊝⊝⊝ Very low certainty.

* Note on numbers: Primary studies are not independent between reviews due to overlap (CCA = 12.3%). The number represents unique studies included in each group of reviews.

** Participants: Some participants may be counted multiple times if they participated in studies included in different reviews.

*** Expanded population: Includes youth and adults with overweight/obesity.

a High heterogeneity (*I*^2^ > 75%) in meta-analyses by Moller et al. (32) and Liu et al. (38).

b 11/20 reviews with low or critically low AMSTAR 2 quality.

c Moderate effect replicated in multiple reviews.

d I^2^ = 81% (26); substantial differences between reviews.

e Predominance of observational studies [33/36 in Lee et al. (31)].

f Effects from null to large between studies.

g Wide CIs; some studies with *n* < 30.

h 9/20 reviews did not adequately consider bias.

i Consistent use of validated accelerometers.

j Heterogeneous measurement protocols.

k All studies consistently report 3–6 METs.

l Calorimetry and direct VO₂ measurement.

m ES > 0.7 for cardiorespiratory fitness.

n Consistent findings between Deng et al. (37), Taylor et al. (25), Zheng et al. (27) and Chen et al. (33).

o Small sample sizes in some studies.

p Clinical relevance for obesity prevention/management.

g Accelerometers in majority of studies Gao et al. (29),, Höchsmann et al. (21),.

r Only 23% of interventions with lasting effects (8).

s < 5 studies per review evaluated >6 months.

t Majority measured only during active intervention.

u Only 30% of reviews systematically reported (6/20).

v Absence of uniform protocols for adverse event collection/reporting.

For the primary outcome of general physical activity levels, evaluated in 20 reviews that included 418 unique primary studies with approximately 19,383 participants, evidence certainty was rated as moderate. On one hand, methodological limitations were substantial, with 11 of the 20 reviews rated as low or critically low quality according to AMSTAR 2. Inconsistency was serious, evidenced by *I*^2^ values consistently superior to 75% in the main meta-analyses, particularly the 87.7% reported by Moller et al. (32). However, these negative factors were partially compensated by the magnitude and consistency of the main effect: a moderate effect size (Hedges g = 0.525, 95% CI: 0.322–0.728) replicated directionally in multiple independent reviews, suggesting a real effect despite variability in its magnitude.

Specific physical activity outcomes showed diverse patterns of certainty. For daily steps, evaluated mainly in the three reviews on Pokémon Go (26, 31, 34) with 63 studies and more than 72,000 participants, certainty was low. This degradation reflected multiple concerns: serious inconsistency manifested in the *I*^2^ = 81% reported by Khamzina et al. (26), the predominance of observational designs (33 of 36 studies in Lee et al., were observational) (31), and the unique nature of Pokémon Go that limits generalization to other AVG. The finding of an average increase of 1,446 daily steps, although statistically robust, must be interpreted with considerable caution given these limitations.

Time in MVPA, evaluated in 14 reviews with 134 studies, also showed low certainty. The degradation was multifactorial: serious inconsistency with effects varying from null to large between studies, significant imprecision with wide confidence intervals and some studies with very small samples (*n* < 30), and substantial risk of bias given that 14 of the 20 reviews did not adequately consider bias in their analyses. Objective measurement through validated accelerometers in most studies provided some additional confidence, slightly elevating the final rating, but not sufficient to reach moderate certainty.

A more robust finding emerged for intensity during gameplay, measured in METs. Based on 7 reviews with 93 studies and almost 5,000 participants, certainty was moderate. Despite limitations from heterogeneous measurement protocols between studies, the notable consistency was striking: all studies converged on a range of 3–6 METs during active gameplay. This convergence, combined with the use of gold standard measurement methods such as indirect calorimetry and direct VO₂ measurement in many studies, substantially increased confidence. This finding suggests that, independent of other factors, AVG reliably achieve at least light to moderate physical activity intensity when played as designed.

The most convincing results emerged in specific populations. For older adults, evidence certainty reached the high level, the highest in the entire UR. This rating was based on 5 reviews with 80 studies totaling 3,355 participants. No factor seriously degraded confidence: studies were predominantly well-designed RCTs, results were consistent between reviews, particularly among (25, 33, 37), and effect sizes were large and clinically significant. Effects on muscle strength (ES = 0.64–0.68) and cardiorespiratory fitness (ES = 0.79) were not only statistically significant but comparable or superior to traditional exercise interventions in this population. Consistency between independent reviews using different inclusion criteria further strengthened confidence.

For people with overweight/obesity (including both youth and adults), certainty was moderate. Based on 6 reviews with 68 studies and 2,279 participants, evidence showed consistent benefits although with some limitations. Imprecision was the main degradation factor, with relatively small sample sizes in individual studies. However, clear clinical relevance for obesity prevention and management, combined with predominant objective measurement through accelerometers and consistency of positive findings between reviews Höchsmann et al. (21), Gao et al. (29), Zhao et al. (36), justified a moderate certainty rating.

Temporal aspects of interventions showed dramatically lower certainty. Sustainability of effects beyond 6 months received a very low certainty rating, the lowest possible in the GRADE system. This evaluation, based on 7 reviews with 58 studies, reflected multiple severe deficiencies. Inconsistency was very serious: only 23% of interventions showed lasting effects according to the seminal analysis by Peng et al. (8), a finding consistently replicated. Imprecision was serious, with fewer than 5 studies per review evaluating long-term follow-up. More problematically, evidence was predominantly indirect, as most studies measured outcomes only during active intervention, requiring extrapolation to infer sustainability.

Similarly, the evaluation of safety and adverse events showed very low certainty. With only 6 of 20 reviews systematically reporting this data, the evidence base was fragmentary at best. The complete absence of standardized protocols for collection and reporting of adverse events in primary studies made any meaningful synthesis impossible. Although available data suggest that serious adverse events are rare, confidence in this conclusion is minimal given inadequate surveillance. This limitation is particularly concerning considering the promotion of AVG for vulnerable populations such as older adults at risk of falls or children with complex medical conditions.

The GRADE evaluation also considered the presence of publication bias, formally evaluated in only 11 of the 20 reviews. Among these, 3 reviews Moller et al. (32), Liu et al. (38), Deng et al. (37) identified evidence of possible bias through asymmetric funnel plots, suggesting that small studies with negative results may be underrepresented in the literature. This possibility contributed to degrading certainty for several outcomes, although the impact was generally moderate given that main findings came from larger studies less susceptible to this bias.

## Discussion

### Main findings

This UR synthesized evidence from 20 SR that evaluated the impact of active video games on physical activity, encompassing 418 unique primary studies with more than 180,000 participants. Our findings reveal a fundamental paradox: while there is consistent evidence that AVG can promote light to moderate intensity physical activity during their use, the extreme methodological heterogeneity between studies makes it impossible to establish specific clinical recommendations or determine the true magnitude of their effectiveness. This situation reflects not so much a lack of intervention efficacy, but rather a systemic failure in research standardization in this emerging field.

### Effectiveness of AVG: what we know with certainty

The most robust evidence indicates that AVG consistently achieve intensities of 3–6 METs during gameplay sessions, equivalent to light-moderate physical activity (8). This consistency across multiple platforms, populations and contexts suggests that, independent of other factors, AVG effectively transform sedentary screen time into physical activity. The most comprehensive meta-analysis identified Moller et al. (32) reported a moderate effect size (Hedges g = 0.525), comparable to other physical activity interventions in pediatric populations (40).

Effectiveness showed important variations according to the population studied. Older adults emerged as the group with the most consistent and largest magnitude benefits, with significant improvements in muscle strength (ES = 0.64–0.68) and cardiorespiratory fitness (ES = 0.79), findings supported by high certainty of evidence according to our GRADE evaluation, although this certainty is primarily applicable to the populations in high-income countries where the vast majority of research has been conducted. These effects are particularly relevant considering that traditional strength training in older adults typically produces similar effect sizes (ES = 0.68) according to previous meta-analyses (41). Notably, independent reviews converged on these positive findings (25, 27, 33, 37), strengthening confidence in these results. For people with overweight and obesity (both youth and adults), AVG showed consistent benefits in six reviews, while in the general pediatric population, effects were more heterogeneous, coinciding with previous reviews that suggest greater effectiveness in populations with lower baseline physical activity (42).

A concerning finding was the limited evidence of sustained effects beyond the intervention period. Only 23% of evaluated interventions showed maintenance of increases in physical activity after finishing the program, a problem consistently identified from the earliest reviews (8) to the most recent (35). This lack of sustainability seriously questions the potential of AVG as a long-term public health strategy and suggests that it might function better as a complement rather than a replacement for other forms of physical activity promotion (43). The Pokémon Go phenomenon perfectly illustrates this challenge. Despite achieving impressive initial increases in daily steps (1,446 steps), multiple studies documented a gradual decline in effect over time (43, 44). This “novelty-decline” pattern has been consistently observed in technological health interventions and suggests the need for specific strategies to maintain long-term engagement (45).

### The central problem: extreme methodological heterogeneity

The most significant problem identified in this UR was not the variable effectiveness of AVG, but the almost total absence of methodological standardization in their evaluation. This heterogeneity manifests in multiple dimensions that make meaningful comparison between studies virtually impossible. Studies used a bewildering variety of metrics: METs, VO₂max, percentage of maximum heart rate, steps per day, minutes in MVPA, kilocalories per minute, among others. This diversity is not merely technical; it represents fundamentally different conceptualizations of what constitutes “effective physical activity.” While some researchers prioritized instantaneous intensity (METs), others focused on accumulated volume (steps/day) or time in specific intensity zones (MVPA), reflecting the lack of conceptual consensus in the field (46).

Variability in measurement methods was equally problematic, from laboratory indirect calorimetry to self-reported questionnaires, through multiple generations and brands of accelerometers with different proprietary algorithms. This situation is analogous to attempting to compare temperatures measured with mercury, digital thermometers and subjective estimates of “heat,” an analogy that illustrates the magnitude of the problem (47). Intervention durations varied from single 60-min sessions to 48-week programs (39), with frequencies from once to 5 times per week, in contexts as diverse as controlled laboratories, schools, homes and public spaces. This variability makes it impossible to determine optimal “doses” or establish specific implementation recommendations, a problem recognized in international physical activity guidelines (5).

As Ramírez-Granizo et al. (30) aptly noted: “very disparate results due to the wide variety of contexts, instruments used, duration and methodologies.” This observation, repeated in multiple reviews, underscores that the problem is not occasional but systemic. The high statistical heterogeneity found (*I*^2^ > 75% in most meta-analyses) is a symptom of this deeper methodological problem, not simply expected clinical variability.

### Implications for clinical practice and urgent need for standardization

With current evidence, we can establish tentative but important recommendations for clinical practice. For older adults in supervised settings within high-income contexts, AVG (particularly Nintendo Wii) can be recommended with confidence as a complement or alternative to traditional exercise, especially for those with barriers to conventional physical activity (48). The convergence of five independent reviews on this finding significantly strengthens this recommendation. For children and adolescents with overweight or obesity, AVG can be a useful motivational tool, although they should not be considered as complete substitute for structured physical activity (49). In school contexts, evidence suggests that AVG can complement but not replace traditional physical education. Their use might be more appropriate on bad weather days, as a structured recreation activity, or for students with limitations in participating in traditional sports.

An important consideration inadequately addressed in the current evidence base is adherence and long-term acceptability of AVG interventions. Only three of the 20 included reviews reported adherence outcomes, and none conducted systematic comparative analyses with conventional exercise (24, 25, 27). Available limited data suggest adherence may be comparable or slightly superior to traditional exercise in supervised settings, though the drivers of sustained engagement remain poorly understood (24). Systematic reviews of digital health interventions suggest that incorporation of self-monitoring, goal-setting, and social support features can enhance long-term adherence to technology-based physical activity programs (45). Future research should systematically examine modifiable factors influencing adherence, including platform usability and accessibility, quality of onboarding and ongoing support, and integration of evidence-based behavior change techniques to counter the “novelty decay” phenomenon we identified (45). Without this evidence, uncertainty about real-world implementation sustainability must tempered enthusiasm for AVG effectiveness during active use.

Effective implementation of AVG in health systems requires considering several critical factors. First, there is a need for health personnel training on the characteristics and limitations of different platforms, as inadequate prescriptions could result in low adherence or suboptimal effects. Second, consider the socioeconomic factors, as equipment costs may represent a significant barrier for vulnerable populations, precisely those who could benefit most from these interventions. Third, integration with other health promotion components, recognizing that AVG function better as part of multicomponent interventions that include nutritional education and behavioral modification (50). The almost total absence of safety data, systematically reported in only 6 of 20 reviews (30%), represents a critical gap that must be urgently addressed before large-scale implementation.

The methodological heterogeneity identified is not merely an academic inconvenience; it represents a fundamental barrier to advancing the field and translating evidence into practice. Without a concerted effort toward standardization, we will continue accumulating evidence that, although individually valid, collectively results incomparable and useless for informing policies or clinical practice. We propose that future research adopt a minimum set of standardized measures that include: time in MVPA measured by accelerometry using validated cut points for the specific population (51), METs measured or estimated using standardized protocols (52), daily steps and total energy expenditure using validated devices (53), follow-up evaluations at 3, 6 and 12 months post-intervention as minimum, and a standardized protocol for adverse event reporting following CONSORT guidelines (54). This standardization is particularly urgent given the rapid technological advancement that continuously introduces new platforms and AVG modalities.

### Health equity implications and the digital divide

While AVGs present a promising opportunity to promote physical activity, their implementation must be considered through a health equity lens to avoid exacerbating existing health disparities. A primary concern is the economic accessibility of AVG technologies and the digital divide they may perpetuate. While some commercial off-the-shelf exergames (e.g., mobile applications, basic motion-tracking games) may cost $20–50 USD plus a compatible device, full console systems with motion sensors range from $200–500 USD, and specialized serious games with proprietary hardware can exceed $1,000 USD, representing months of income in low- and middle-income contexts (43). Beyond initial purchase costs, ongoing expenses including software updates, subscription services, reliable internet connectivity requirements, and device replacement create sustained financial barriers (43, 55). These costs can be prohibitive for low-income families and vulnerable populations, precisely those who might benefit most from accessible physical activity interventions.

The cost differential between COTS exergames (broadly accessible) and serious games (potentially more effective but prohibitively expensive for individuals and under-resourced health systems) creates a troubling paradox: populations that might benefit most from structured, evidence-based serious games are least able to afford them, while accessible COTS options may lack the behavioral architecture necessary for sustained effectiveness. The overwhelming concentration of research in high-income countries, as demonstrated in this review, further compounds this issue, leaving a critical evidence gap on the feasibility and effectiveness of AVGs in resource-limited settings where implementation barriers may be most pronounced.

Furthermore, the cultural relevance of commercially available AVGs is a significant yet under-studied factor (55). Most games are developed in and for North American, European, or East Asian markets, and may lack the cultural resonance needed to engage diverse populations, including those in Latin America, Africa, or South Asia. Language barriers, representation in game content, and culturally specific movement patterns or activity preferences are rarely considered in mainstream commercial platforms. For AVG interventions to be truly effective globally, they must be appealing and culturally appropriate for the communities they aim to serve.

Future research and public health initiatives must prioritize inclusive implementation. Strategies could include placing AVG stations in publicly accessible locations like community centers and schools in underserved areas, exploring low-cost technological solutions and open-source platforms, and employing co-design methods to develop culturally tailored games with direct input from target communities (55). Without a deliberate focus on equity, AVGs risk becoming a tool that benefits only the most privileged, widening the very health gaps they have the potential to close.

## Strengths, limitations and future directions

This UR presents several important methodological strengths. It represents the most comprehensive synthesis to date on AVG and physical activity, including 20 SR published until 2025 and applying systematic quality assessment with validated tools (AMSTAR 2 and GRADE). The inclusion of reviews in multiple languages and the search in grey literature increases the comprehensiveness of our findings. The rigorous application of the GRADE system with differentiated evaluation by population and outcome provides a nuanced picture of evidence certainty. However, we recognize important limitations. The high overlap between primary studies (CCA = 12.3%) may have inflated some estimates, although we attempted to minimize this effect by prioritizing the most recent and comprehensive reviews. A major limitation of the current evidence base is the predominance of studies from high-income countries, severely restricting our findings' global generalizability. As such, our conclusions—including the high certainty of evidence for older adults—must be interpreted with caution, as they may not be applicable to middle- and low-income contexts where technological access and cultural factors differ. Finally, the review protocol was not registered *a priori*. While pre-registration is an established standard for systematic reviews, its application for umbrella reviews is an evolving practice. For this reason, formal registration was not undertaken by the authors. To compensate for this limitation and ensure methodological rigor, the study was strictly conducted based on a pre-specified internal protocol following JBI and PRIOR guidelines, and all steps have been meticulously reported for full transparency and reproducibility.

Looking toward the future, we identify several critical priorities for research. Beyond urgent methodological standardization, studies that specifically examine mechanisms of behavioral change maintenance in AVG are needed, possibly incorporating evidence-based behavior change techniques and contemporary psychological theories (55). Research in middle and low-income countries is particularly crucial, not only to improve generalizability but also because these contexts may offer unique approaches on implementation under resource-limited conditions. Implementation studies that examine AVG integration in existing health and education systems are essential to translate efficacy evidence into real population impact. Finally, research on emerging technologies such as immersive virtual reality, brain-computer interfaces and adaptive games with artificial intelligence represents a promising frontier that could overcome the sustainability limitations identified in current AVG generations (56).

## Conclusions and recommendations

In conclusion, this umbrella review of 20 systematic reviews confirms that active video games can be effective tools for promoting physical activity during their use, with particularly strong evidence in older adults and people with overweight within the context of the high-income countries where this evidence was generated. The convergence of multiple independent reviews on these findings significantly strengthens confidence in these specific populations. However, three critical barriers limit translation of this evidence into actionable recommendations: the lack of methodological standardization in outcome measurement and intervention protocols, the systematic underreporting of adverse events in only 30% of reviews, and the near-complete absence of evidence from low- and middle-income settings. The methodological heterogeneity identified—with studies employing incomparable metrics (METs, MVPA, steps/day), diverse measurement instruments (accelerometers with different algorithms, calorimetry, questionnaires), and vastly different protocols (single sessions to 48-week programs)—prevents synthesis of specific dosage recommendations or identification of optimal implementation strategies. Until the research community adopts standardized core outcome measures and reporting protocols, the true potential of AVGs as a public health tool will remain unrealized.

Current evidence justifies the cautious use of AVGs in specific populations, particularly older adults in supervised settings and individuals who are overweight and seeking alternatives to traditional exercise. However, the incomplete safety profile requires vigilant monitoring for adverse events, including fall risk, cybersickness, and musculoskeletal injuries. However, realizing the full potential of these technologies in public health promotion requires a coordinated international effort toward methodological standardization, comprehensive adverse event reporting, and inclusive research that addresses the economic and cultural barriers limiting equitable access globally. The urgency of addressing this situation cannot be underestimated, especially considering AVGs' potential to tackle the global crisis of physical inactivity and the rapid technological advancements that continue to expand intervention possibilities.
