# Supplementary material for: Effectiveness of active video games for promoting physical activity: an umbrella review
Source: Front Sports Act Living. 2025 Nov 17;7:1706145. doi: 10.3389/fspor.2025.1706145 (PMC12666624; doi:10.3389/fspor.2025.1706145)
Supplement: Supplementary file 1 [file Datasheet1.pdf]

Supplementary material 1. Search strategy

PubMed

Strategy	Search terms
#1	Video Games"[Mesh] OR "Exergaming"[Mesh] OR exergam*[tiab] OR "active video game*"[tiab] OR "serious game*"[tiab] OR "motion-based video game*"[tiab] OR "Nintendo Wii"[tiab] OR "Wii Fit"[tiab] OR "Dance Dance Revolution"[tiab] OR "DDR"[tiab] OR "Xbox Kinect"[tiab] OR "Kinect"[tiab] OR "Pokémon Go"[tiab] OR "Pokemon Go"[tiab]
#2	"Exercise"[Mesh] OR "Motor Activity"[Mesh] OR "Physical Fitness"[Mesh] OR "physical activit*"[tiab] OR "exercise"[tiab] OR "motor activit*"[tiab]
#3	systematic[sb] OR "systematic review"[tiab] OR "systematic reviews"[tiab] OR meta-analysis[pt] OR meta-analys*[tiab] OR "umbrella review"[tiab] OR "overview of reviews"[tiab]
#4	#1 AND #2 AND #3

Scopus

Strategy	Search terms
#1	TITLE-ABS-KEY (exergam* OR "active video game*" OR "motion-based video game*" OR "serious game*" OR "Nintendo Wii" OR "Wii Fit" OR "Dance Dance Revolution" OR "DDR" OR "Xbox Kinect" OR kinect OR "Pokémon Go" OR "Pokemon Go")
#2	TITLE-ABS-KEY ("physical activity" OR "physical activities" OR exercise OR "motor activity" OR "physical fitness")
#3	TITLE-ABS-KEY ("systematic review" OR "systematic reviews" OR meta-analysis OR meta-analyses OR "meta analysis" OR "umbrella review" OR "overview of reviews")
#4	#1 AND #2 AND #3

Web of Science

Strategy	Search terms
#1	TS=(exergam* OR "active video game*" OR "motion-based video game*" OR "serious game*" OR "Nintendo Wii" OR "Wii Fit" OR "Dance Dance Revolution" OR DDR OR "Xbox Kinect" OR Kinect OR "Pokémon Go" OR "Pokemon Go")
#2	TS=('physical activity'/exp OR 'motor activity'/exp OR 'exercise'/exp OR 'physical fitness'/exp)
#3	TS=('systematic review':ti,ab,kw OR 'systematic reviews':ti,ab,kw OR 'meta analyses':ti,ab,kw OR 'meta analysis':ti,ab,kw OR 'umbrella review':ti,ab,kw OR 'overview of reviews':ti,ab,kw)
#5	#1 AND #2 AND #3

Embase

Estrategia	Search terms
#1	'exergaming'/exp OR 'active video game'/exp OR exergam*:ti,ab OR 'nintendo wii':ti,ab OR 'pokemon go':ti,ab)
#2	'physical activity'/exp OR 'motor activity'/exp OR 'exercise'/exp OR 'physical fitness'/exp
#3	('systematic review':ti,ab,kw OR 'systematic reviews':ti,ab,kw OR 'meta analyses':ti,ab,kw OR 'meta analysis':ti,ab,kw OR 'umbrella review':ti,ab,kw OR 'overview of reviews':ti,ab,kw)
#5	#1 AND #2 AND #3

Epistemonikos

Strategy	Search terms
#1	exergaming OR "active video game" OR "serious game" OR "Nintendo Wii" OR "Wii Fit" OR "Dance Dance Revolution" OR "Xbox Kinect" OR "Pokemon Go"
#2	Filter: Systematic Review
#3	#1 AND #2

LILACS/BVS

Strategy	Search terms

#1	tw:(exergam* OR "videojuegos activos" OR "juegos activos" OR "videojuegos de movimiento" OR "Nintendo Wii" OR "Wii Fit" OR "Dance Dance Revolution" OR "DDR" OR "Xbox Kinect" OR "Kinect" OR "Pokémon Go" OR "Pokemon Go")
#2	tw:("actividad física" OR ejercicio OR "aptitud física" OR "actividad motora" OR "ejercicio físico"))
#3	#1 AND #2

Supplementary Material 2. Detailed AMSTAR 2 assessment of included systematic reviews

Author/Year	Item 1	Item 2*	Item 3	Item 4*	Item 5	Item 6	Item 7*	Item 8	Item 9*	Item 10	Item 11*	Item 12	Item 13*	Item 14	Item 15*	Item 16	Overall Rating
Norris et al., 2016	✓	✗	✓	✓	✓	✓	✗	?	✓	✗	N/A	N/A	✗	✓	✗	✓	**Low**
Peng et al., 2013	✓	✗	✓	✓	✓	✓	✗	✓	?	✗	✓	✓	✗	✓	✗	✓	**Low**
Höchsmann et al., 2015	✓	✗	✓	✓	✓	✓	✗	✓	✓	✗	✓	✓	?	✓	?	✓	**Moderate**
Khamzina et al., 2019	✓	✗	✓	✓	✓	✓	✗	✓	✓	✗	✓	✓	?	✓	?	✓	**Moderate**
Street et al., 2017	✓	✗	✓	✓	✓	✓	✗	?	?	✗	N/A	N/A	✗	?	✗	✓	**Low**
Zheng et al., 2019	✓	✗	✓	✓	✓	✓	✗	✓	✓	✗	N/A	N/A	?	✓	✗	✓	**Low**
Williams et al., 2020	✓	✗	✓	✓	✓	✓	✗	?	✓	✗	N/A	N/A	✗	✓	✗	✓	**Low**
Gao et al., 2020	✓	✗	✓	✓	✓	✓	✗	✓	✓	✓	✓	✓	✓	✓	✓	✓	**Moderate**
Ramírez-Granizo et al., 2020	✓	✗	?	?	✓	✓	✗	✗	✗	✗	N/A	N/A	✗	?	✗	?	**Critically low**
Lee et al., 2021	✓	✗	✓	✓	✓	✓	✗	✓	✓	✗	N/A	N/A	?	✓	✗	✓	**Low**
Moller et al., 2023	✓	✓	✓	✓	✓	✓	✓	✓	✓	✓	✓	✓	✓	✓	✓	✓	**High**
Chen et al., 2023	✓	✓	✓	✓	✓	✓	?	✓	✓	✓	✓	✓	✓	✓	✓	✓	**High**
Liang et al., 2023	✓	✗	✓	✓	✓	✓	✗	?	✓	✗	N/A	N/A	?	✓	✗	✓	**Low**
Zhao et al., 2024	✓	✗	✓	✓	✓	✓	✗	✓	✓	✗	N/A	N/A	✗	✓	✗	✓	**Low**
Deng et al., 2024	✓	✓	✓	✓	✓	✓	?	✓	✓	✓	✓	✓	✓	✓	✓	✓	**High**
Liu et al., 2024	✓	✓	✓	✓	✓	✓	✓	✓	✓	✓	✓	✓	✓	✓	?	✓	**Moderate**
Spring et al., 2025	✓	✗	✓	?	✓	✓	✗	?	?	✗	N/A	N/A	✗	?	✗	✓	**Critically low**
Lamas et al., 2023	✓	✗	✓	✓	✓	✓	✗	?	?	✗	N/A	N/A	✗	?	✗	?	**Critically low**
Pakarinen et al., 2016	✓	✗	✓	✓	✓	✓	✗	✓	✓	✗	✓	✓	?	✓	✗	✓	**Moderate**
Taylor et al., 2018	✓	✓	✓	?	✓	✓	✗	✓	✓	✓	✓	✓	✓	✓	?	✓	**Moderate**

Legend: ✓ = Yes (fully meets); ✗ = No (does not meet); ? = Partial (partially meets); N/A = Not applicable (did not perform meta-analysis) *Critical items

AMSTAR 2 item descriptions:

- Item 1: PICO components
- Item 2*: Protocol registered a priori
- Item 3: Explanation of included designs
- Item 4*: Comprehensive search strategy
- Item 5: Duplicate selection
- Item 6: Duplicate extraction
- Item 7*: List of excluded studies with justification
- Item 8: Adequate description of included studies
- Item 9*: Risk of bias assessment
- Item 10: Funding sources of included studies
- Item 11*: Appropriate meta-analytical methods
- Item 12: Impact of risk of bias on meta-analysis
- Item 13*: Consideration of risk of bias in interpretation
- Item 14: Explanation of heterogeneity
- Item 15*: Publication bias assessment
- Item 16: Conflict of interest statement

Supplementary Table 3. ROBIS assessment for reviews with predominance of non-randomized studies

Author/Year	Domain 1: Eligibility criteria	Domain 2: Identification and selection	Domain 3: Collection and evaluation	Domain 4: Synthesis and findings	Overall risk of bias
Peng et al., 2013[1]	Low	Low	High	High	**High**
Khamzina et al., 2019[2]	Low	Low	Unclear	High	**High**
Street et al., 2017[3]	Unclear	Low	High	High	**High**
Ramírez-Granizo et al., 2020[4]	High	Unclear	High	High	**High**
Lee et al., 2021[5]	Low	Low	Low	Unclear	**Unclear**
Zheng et al., 2019[6]	Low	Low	Unclear	Unclear	**Unclear**
Liang et al., 2023[7]	Low	Low	Unclear	Unclear	**Unclear**
Lamas et al., 2023[8]	Unclear	Unclear	High	High	**High**

Notes:

- [1]28 of 41 studies were observational laboratory studies
- [2]16 of 17 studies were observational
- [3]Included mixed studies with significant observational component
- [4]Included mixed longitudinal and experimental studies

- [5]33 of 36 studies were observational
- [6]Included studies with frail populations, mixed designs
- [7]Included multiple designs: qualitative, cross-sectional and cohorts
- [8]11 of 26 studies were quasi-experimental

Justification for main ratings:

- **Domain 1**: High risk when inclusion criteria unclear or too broad
- **Domain 2**: High risk when search limited or not exhaustive
- **Domain 3**: High risk when quality tools inadequate or not consistently applied
- **Domain 4**: High risk when synthesis inappropriate for study types or without quality consideration
